# Dynamic sampling of liquid metal structures for theoretical studies on catalysis†

**DOI:** 10.1039/d3sc04416e

**Published:** 2023-11-29

**Authors:** Charlie Ruffman, Krista G. Steenbergen, Anna L. Garden, Nicola Gaston

**Affiliations:** a MacDiarmid Institute for Advanced Materials and Nanotechnology, Department of Physics, University of Auckland Private Bag 92019 Auckland New Zealand n.gaston@auckland.ac.nz; b MacDiarmid Institute for Advanced Materials and Nanotechnology, Department of Physics, School of Chemical and Physical Sciences, Victoria University of Wellington PO Box 600 Wellington 6140 New Zealand; c MacDiarmid Institute for Advanced Materials and Nanotechnology, Department of Chemistry, University of Otago P.O. Box 56 Dunedin 9054 New Zealand

## Abstract

Liquid metals have recently emerged as promising catalysts that can outcompete their solid counterparts for many reactions. Although theoretical modelling is extensively used to improve solid-state catalysts, there is currently no way to capture the interactions of adsorbates with a dynamic liquid metal. We propose a new approach based on *ab initio* molecular dynamics sampling of an adsorbate on a liquid catalyst. Using this approach, we describe time-resolved structures for formate adsorbed on liquid Ga–In, and for all intermediates in the methanol oxidation pathway on Ga–Pt. This yields a range of accessible adsorption energies that take into account the at-temperature motion of the liquid metal. We find that a previously proposed pathway for methanol oxidation on Ga–Pt results in unstable intermediates on a dynamic liquid surface, and propose that H desorption must occur during the path. The results showcase a more accurate way to treat liquid metal catalysts in this emerging field.

## Abstract 2

*Ab initio* molecular dynamics sampling of an adsorbate on a liquid catalyst yields a more detailed treatment for catalysis.

## Graphical abstract

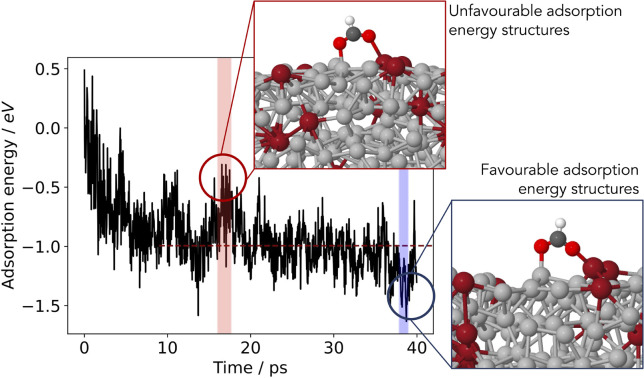

## Introduction

A new group of catalysts that have recently emerged in both the experimental and computational literature are low temperature liquid metals.^1–5^ These promising materials are typically characterised as pure metals or alloys which melt at notably lower temperatures than the majority of metals in the periodic table (often <330 °C).^3^ Recent high-profile reports of their catalytic activity include systems such as supported liquid Pd–Ga phases which are highly active and selective for butane dehydrogenation,^6^ molten Ni–Bi for the pyrolysis of methane to produce H_2_,^4^ and a liquid galinstan–Ce alloy for the reduction of CO_2_ to solid carbon species.^7^

Liquid metals can offer unique advantages over traditional solid-phase heterogeneous catalysts. For instance, solid metal catalysts are known to suffer from problems such as surface coking which can deactivate the material and shorten its lifetime.^2,8^ In contrast, liquid metals are more resilient to by-product build up, and can replenish their surface,^9^ giving rise to what is often termed “self-healing” behaviour.^2,6^

Some liquid metal catalysts have shown fascinating differences in activity to their solid counterparts. Sometimes these differences manifest as greater activity (*e.g.* liquid Ga–Pt is three orders of magnitude more active towards methanol oxidation than solid Pt),^10^ or other-times as selectivity differences (*e.g.* solid Ga–Sn is inactive to CO_2_ reduction, but the liquid produces formate with 95% faradaic efficiency).^11^ The unprecedented catalytic activity of these liquid metals, and their promising regenerative behaviour, make them a highly attractive advance in the long-standing field of heterogeneous catalysis.

To date, the vast majority of past work on liquid metals has been experimental in nature, though some density functional theory (DFT) studies have provided additional evidence to support behaviour that has been observed *in situ*.^12,13^ In contrast, for solid catalysts, DFT calculations are used extensively in areas such as screening for new catalysts,^14,15^ mapping adsorption energies and reaction paths to optimise current catalysts,^16,17^ as well as for determining reaction mechanisms, energy barriers, and reaction rates.^18–20^ When applying DFT to solid heterogeneous catalysts, it is typical to take structures that are at energy minima and at 0 K,^17,21,22^ calculating energies of intermediate states based on the interaction energy between this static surface and the adsorbates. While this may be a sensible assumption for a solid structure, trying to apply these standard DFT methods to liquid metal catalysts proves challenging, as they are unable to account for the dynamic nature of an “at-temperature” liquid metal surface. Additionally, there is also evidence of significant mobility in the atoms of solid metal surfaces^23^ and in nanoparticles.^24^ These dynamics are thought to be important to metal surface reconstruction, but also in catalysis.^25^ For instance, the transient formation of single-atom active sites in gold nanoparticles has been shown to be highly important to catalytic carbon monoxide oxidation.^26^ Therefore, an approach to capture dynamic surface reactions would be valuable primarily to liquid metal catalysts, but also to solid surface catalysts too.

Prior DFT work on liquid metals has used static snapshots – either structures from molecular dynamics trajectories^10,27^ or pristine surface cut analogues^12,13,28,29^ – to probe quantities such as adsorption energies of intermediates. While this is likely a good starting point, we suggest these static adsorption configurations are not sufficient to represent a liquid material, where surface atoms will constantly be undergoing motion and exchange. For instance, the liquid can no longer be considered to be at a static energy minimum, and will instead be sampling structures that are energetically accessible at a given temperature.

Capturing the liquid metal dynamics is particularly important when studying catalysis, as the instantaneous structure of the catalyst likely affects the stability of intermediates. For example, intermediates may preferentially adsorb when the liquid surface is in a favourable configuration (*e.g.* when an active site is present), and react or desorb only once the liquid surface has rearranged (*e.g.* when the adsorption site disappears due to rearrangement, or another active site appears). It is already known that small differences to adsorption energies on solid catalysts (*e.g.* 0.2 eV) can result in reaction rates that differ by orders of magnitude during catalysis. This has previously been observed with single active sites in low abundance dominating reactivity for processes such as ammonia production^30^ or hydrogen evolution.^31^ Therefore, we propose that one of the key factors limiting the DFT modelling of liquid metal catalysts is the absence of thorough methodology to capture the evolution of active sites on a dynamic liquid surface at-temperature.

In this work, we explore a framework by which to study catalysis on liquid metals while capturing the dynamic behaviour of the surface. We employ Vienna *Ab initio* Simulation Package (VASP) molecular dynamics simulations with Perdew–Burke–Ernzerhof for solids (PBEsol) and Perdew–Burke–Ernzerhof (PBE) exchange–correlation functionals to first sample accessible adsorption geometries, then select key regions in time where the adsorption energy is either persistently high or low. Overall, this yields a far more realistic description of adsorption to a dynamic catalyst where at-temperature motion and exchange of atoms is taking place at the adsorption site.

## Results and discussion

### Sampling adsorption geometries

*Ab initio* molecular dynamics (AIMD) is used to concurrently sample the geometry of an adsorbate on the catalyst, and the geometry of the liquid surface below. To achieve this, an AIMD run within the NVT ensemble is initiated from a starting adsorbate geometry on the liquid metal, and allowed to propagate for a certain sampling duration. Full computational details can be found in the ESI.† In most of the samples a 40 ps duration was used. Furthermore, extending this sampling time to 100 ps was not found to locate any structures with significantly higher or lower energy than those found in the first 40 ps (Fig. S1 in the ESI†). In all calculations performed here, first 10 ps of the AIMD simulation are excluded to minimise any bias generated by the starting structure, and to allow the adsorbate to find more favourable arrangements. The unbiased sampling yields a set of adsorbate/liquid metal structures that represent realistic configurations at temperature. This is in contrast methodologies such as metadynamics that sample along prescribed collective variables.^32–34^ Though metadynamics has previously been applied to reactions on surfaces with some dynamic rearrangement,^35,36^ it could not used for a fully liquid metal system until after the surface geometries (and thus possible collective variables for reaction) had been sampled.

To calculate adsorption energies, a suitable reference state must be chosen for the adsorption configurations sampled by the AIMD simulation. For adsorption to solid surfaces, it is customary to use the energy of the clean solid plus the energy of the free adsorbed species as a reference, so that the adsorption energy can be calculated following eqn (1):

1 Δ*E*_ads_ = *E*_(surface+adsorbate)_ − (*E*_surface_ + *E*_adsorbate,free_)

**Fig. 1:**
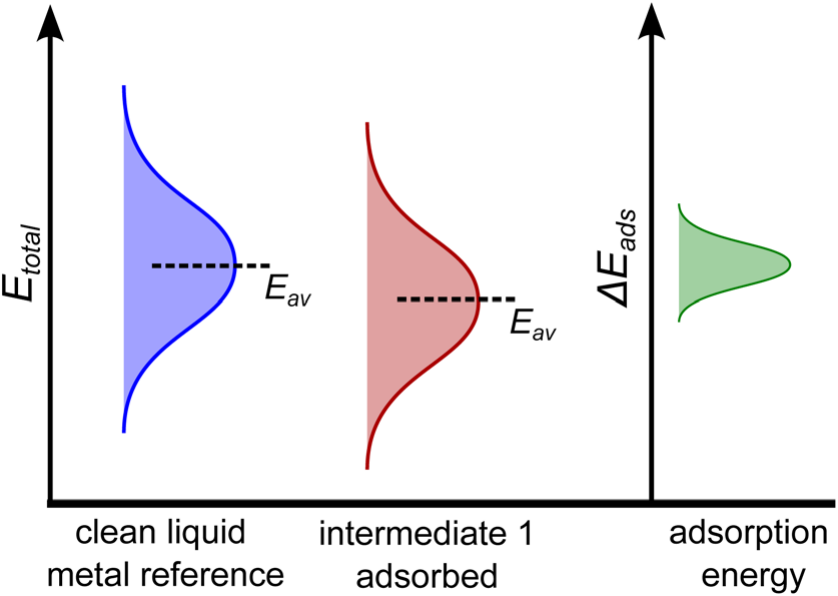
An example schematic showing sampled energies of an at-temperature clean liquid metal (blue), and a liquid metal with the adsorbate present (red). In this example, the adsorbed state is, on average, more stable than the clean liquid metal. The calculated adsorption energies also have a range, denoted by the green curve. Note that, for clarity, the energy of the adsorbate in a box (which is a constant) has been added to the blue curve so that each system has the same atoms accounted for.

From this electronic energy starting point, any desired additive corrections can be applied (*e.g.* solvent corrections).^37^ However, in the case of AIMD sampling of a liquid catalyst, taking a single reference energy for *E*_surface_ is not suitable, as significant structural rearrangement of the catalyst surface occurs during the AIMD simulation that can cause energy differences of several eV. Indeed, it is more suitable to think of the energy of the clean surface as an at-temperature normally-distributed ensemble of energies, and the same is true for the adsorbed state. This idea is schematically shown in Fig. 1 for a hypothetical one-step adsorption process. In this example, the energy of the adsorbed state is shown as lower (on average) than that of the clean reference, suggesting adsorption may be favourable. However, this will not be true when comparing between each individual configuration. For instance, if one compared a particularly low energy structure of the clean surface to the average energy of the adsorbed structure, one would mistakenly conclude this adsorption is unfavourable.

In order to probe the energy of just the adsorbate–surface interaction, and remove any effect of comparing different structures, the geometry of the liquid metal must be the same when calculating the *E*_(surface+adsorbate)_ and the *E*_surface_ terms. This is accomplished by selecting snapshots from the AIMD sampling trajectory every *n* steps, removing the adsorbate, and calculating the single-point energy of the liquid surface in that structure alone. This gives us the energy distribution of the clean liquid metal in Fig. 1. The adsorption energy is then computed on a point-by-point basis, using this adaptive reference for *E*_surface_. The resulting adsorption energy distribution (green curve in Fig. 1) describes the energy differences between clean and adsorbed structures.

The sampling resolution, given by *n*, is chosen as a balance between computational tractability and the temporal precision of adsorption energies. In testing different values for *n* for an adsorbate on a Ga–In alloy liquid (see Fig. S2 to S6 in the ESI†), we found that calculating a reference every 80 fs was sufficient for identifying persistent regions of high or low adsorption energy, as these occurred at the same energy as for finer resolutions (*e.g.* 10–40 fs). Indeed, even loosening this resolution to 100 fs was only associated with relatively small (*e.g.* 0.1 eV) shifts to the maximum and minimum adsorption energies. The average energy remained unchanged in all cases. Here, we have selected an 80 fs resolution, though we note this could be finer or coarser depending on the desired application.

**Fig. 2:**
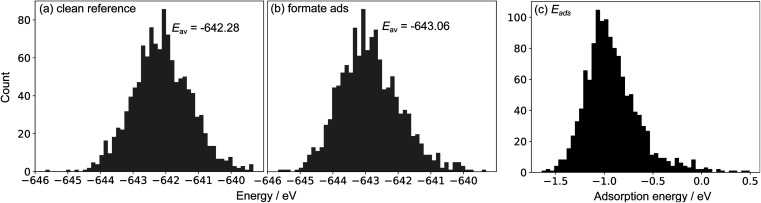
Sample energy distributions for (a) clean Ga–In at 450 K (standard deviation: 0.76 eV). Note that a reference energy of 
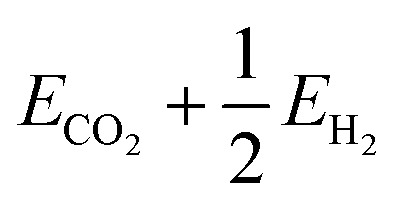
 has been added to allow direct comparison to the formate-adsorbed surface. (b) The energy distribution for Ga–In with formate adsorbed to the surface at 450 K (standard deviation: 0.86 eV). (c) The adsorption energy of formate calculated by subtracting clean energies from adsorbed energies in a matching point-by-point fashion.

To showcase this process for a single-step adsorption of formate (CHOO^−^) onto a Ga–In liquid metal, the relevant energy distributions are depicted in Fig. 2. To keep consistency with past work, the energy of formate in a box is calculated as: 
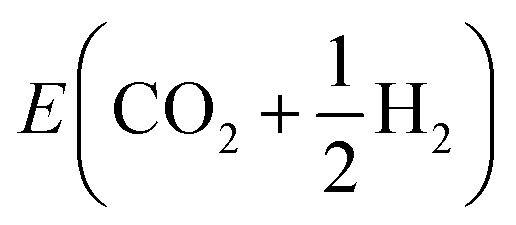
.^38^ It is observed that the distribution for the formate adsorbed structures is shifted down in energy from the clean reference, indicating favourable adsorption. Indeed, when adsorption energies are calculated using matched structures from the two distributions, we observe a broad range of possible adsorption energies (from approximately −1.5 to 0.5 eV), but the majority are closer to the average of −0.99 eV. Any of these possible geometries are spontaneously accessible at the simulated temperature, and therefore should be considered when representing the dynamic liquid metal.

Adsorption of CO_2_ to Ga–In was also considered for this test, but it did not stably bind to the dynamic surface and interact over time. This is consistent with past static relaxations we have performed where a geometry for adsorbed CO_2_ could not be converged. The adsorbate instead leaves the surface. Past work has indicated that proton-assisted adsorption of CO_2_ to make formate may occur on selected elements,^38^ and our results indicate this would be the case on Ga–In liquids.

At this point, it should be noted that we have reported electronic energies of adsorption. For solid-catalyst materials it is possible to estimate entropy corrections from normal mode analysis of a single structure at 0 K.^21,39^ This traditional method can only be applied to liquid systems if one takes a specific snapshot and relaxes it. For instance, for the lowest energy structure of formate on Ga–In, a vibrational entropy correction of *TS*_vib_ = 0.42 eV is calculated at 450 K. However, determining an analogous quantity in liquid metal sampling is complicated by the fact that at-temperature structures are used, and that the entropy change of the surface cannot be neglected during adsorption (which is assumed for solids). Instead, the configurational entropy is inherently included in the molecular dynamics sampling, controlling which energetic states a system is likely to occupy. Therefore, to estimate the entropy of the adsorbate in this dynamic context, we opt to take a configurational entropy (*S*) calculated from the Gibbs entropy formula:2
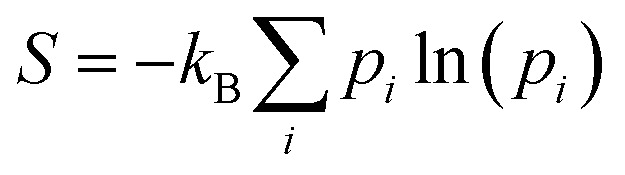
where *p*_*i*_ is the probability of finding the system in a microstate, *i*, with a given configurational energy, *V*_*i*_.

We computed the configurational entropy of three systems at 450 K: (1) a clean Ga–In surface (to serve as a reference, *TS*_clean_), (2) a constrained Ga–In surface where only the formate adsorbate was free to move (*i.e.* all configurational entropy contributions were from the adsorbate alone, *TS*_constrained_), and (3) the fully dynamic Ga–In system with formate adsorbed (where the entropy was a product of surface, adsorbate, and their interactions, *TS*_dynamic_). We found that *TS*_clean_ = 0.307 eV, *TS*_constrained_ = 0.253 eV, and *TS*_dynamic_ = 0.313 eV. The surface-constrained entropy (*TS*_constrained_) is likely most comparable to the solid surface catalysis treatment, where a relaxed structure is taken. Here we find these two values are of the same order of magnitude but with *TS*_constrained_ being 0.17 eV less than *TS*_vib_. This suggests that some differences can be expected between the two treatments of entropy, but that these should be fairly minor if the chosen methodology is applied consistently through reaction pathways.

To calculate the entropy added by the adsorbate in a fully dynamic liquid system, we subtract the entropy of the clean reference (*TS*_clean_) from that of the combined system (*TS*_dynamic_) and find a *T*Δ*S* of 0.00548 eV. While this is a lot smaller than the calculated corrections for the solid, we believe it is realistic. For a liquid, the surface is allowed to respond and compensate to changes in the adsorbate configuration (and *vice versa*). Therefore, the total energy range accessible to the system as a whole is not greatly broadened by the addition of a small surface adsorbate, and the entropy is not greatly increased. As a result, we argue that the entropic corrections to an adsorbate on a liquid metal are not likely to be significant beyond the degree to which they are naturally included in molecular dynamics sampling. Indeed, in exploring these entropy corrections for a different system (methanol adsorbed on liquid Ga–Pt) we observed a similarly small *T*Δ*S* of 0.00286 eV, leading us to conclude that corrections to entropy are negligible regardless of the specific surface intermediate in a reaction path. As a result, the entropy corrections are not included for the subsequent liquid metal systems studied here.

### Selecting notable energy regions

The range of adsorption energies generated over a time-window of sampling raises the question as to what configurations and adsorption energies are catalytically relevant. We argue that one should consider persistent regions of high or low adsorption energy (*i.e.* where the adsorbate is unstable or stable, respectively), in addition to the average adsorption energy over the duration of sampling.

**Fig. 3:**
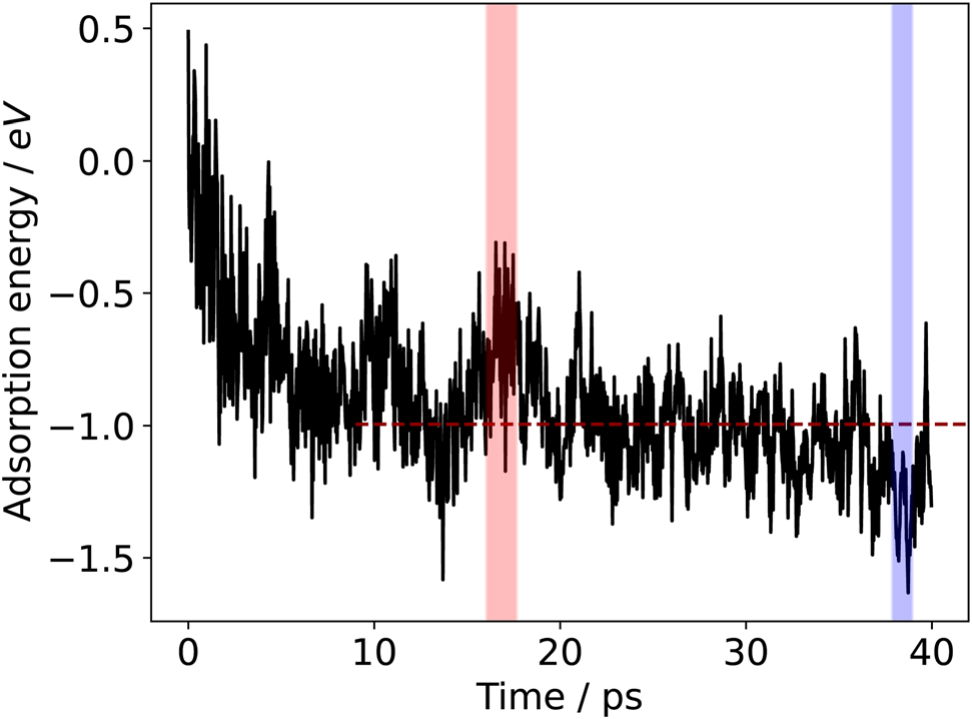
Sampled adsorption energy profile for formate adsorbing to liquid Ga–In at 450 K. The shaded blue region shows a selection of energies that are consistently lower than the average, and the red regions show energies that are consistently higher. The dashed red line indicates the average adsorption energy after 10 ps of equilibration, which is −0.99 eV for this example. The average of the lowest energy region is −1.28 eV and the highest region is −0.63 eV.

The adsorption energy can be plotted over the duration of the AIMD sampling simulation, which is shown for the case of formate adsorbing to a liquid Ga–In surface at 450 K in Fig. 3. A high degree of variability in the adsorption energy is observed, much of which is due to the movement of the adsorbate on the liquid surface. Formate may be transiently arranged in a favourable or unfavourable configuration across snapshots in the simulation, giving rise to the oscillatory noise between snapshots in the plot. However, for catalytic information, regions where the adsorbate is consistently more stable (or less stable) than the average for several picoseconds of time will represent catalyst surface geometries that are more (or less) able to bind surface species. One factor to consider here is that the motion of the liquid metal atoms is slower than that of the surface adsorbate (see Fig. S7 in the ESI† for an analysis of mean squared displacement showing this difference). This difference is partly because the metal atoms are in a bonding network in the metal slab, but also because these nuclei are heavier than those in the adsorbate.

As was previously shown, the adsorption energies are approximately normally distributed over time (Fig. 2). Therefore, we opt to label a region as either stable adsorption (low energy) or unstable adsorption (high energy) based on whether a certain proportion of the adsorption energies are at least some fraction of a standard deviation either below or above the mean for a given duration. Fig. 3 shows the calculated adsorption energy over time for formate on liquid Ga–In at 450 K. The high and low energy regions in the figure were selected by 75% of the energies being at least 0.7 standard deviations away from the mean for at least 1 ps. In this case, we chose the criteria such to select only a single stable and unstable adsorption region over the 40 ps sampling duration. Critically, the requirement of the region persisting for at least 1 ps was implemented so as not to count local transient peaks or troughs in the adsorption energies sampled. Energy regions that deviate from the mean for over 1 ps, with structures occurring sequentially in time, are more likely to represent unique surface configurations that are either favourable or unfavourable. More than one region could also be selected, especially if a long sampling duration is used.

**Fig. 4:**
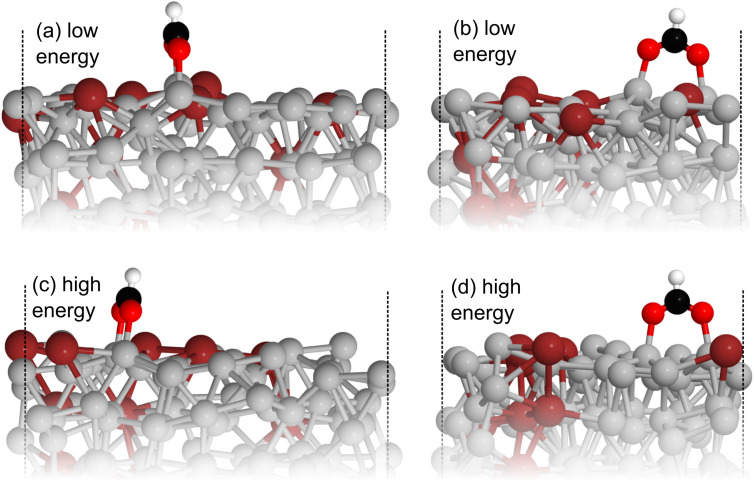
(a) and (b) show side views of the most favourable structure from the more stable region sampled for formate adsorption on liquid Ga–In at 450 K. (c) and (d) show the same views for the analogous structure from the less stable high energy region. It can be seen that the Ga atoms which formate binds to are elevated in the low energy region compared to the high. Key: Ga-silver, In-dark red, O-red, C-black, H-white.

**Fig. 5:**
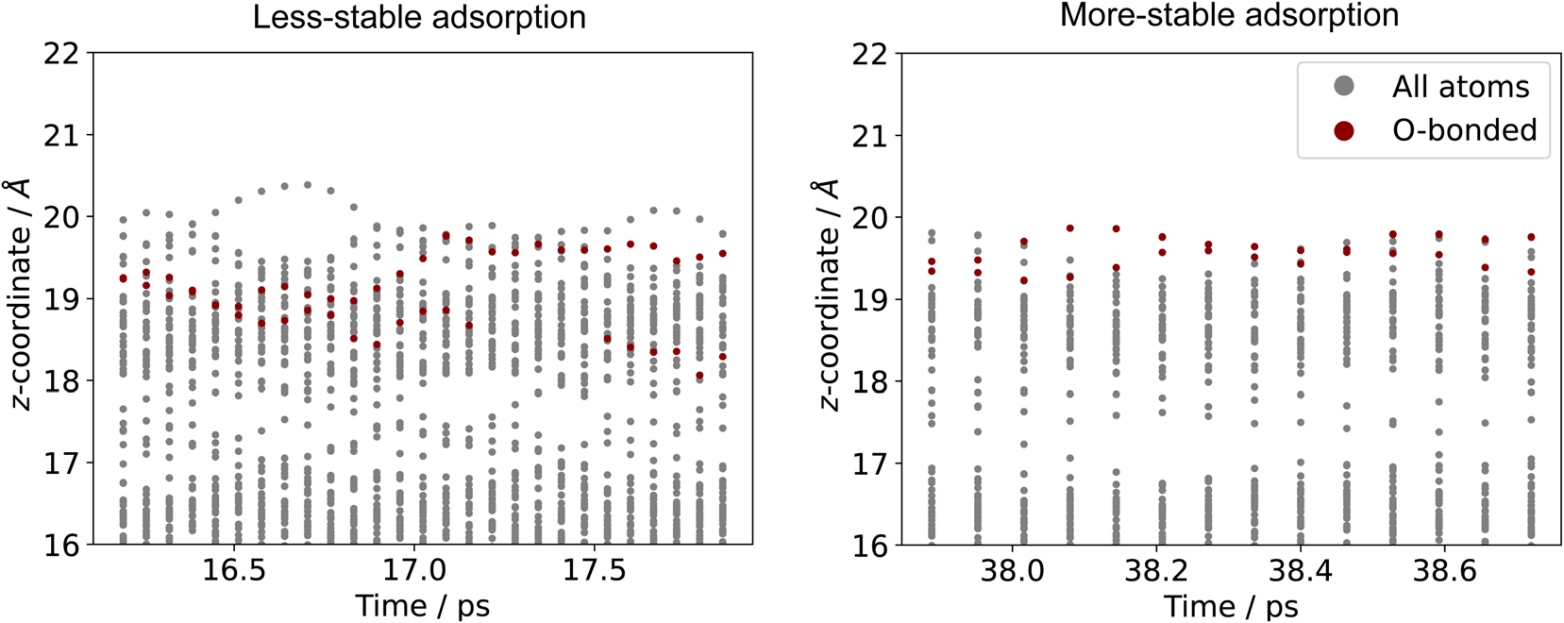
The *z*-coordinates of metal atoms in the surface layers of Ga–In with formate adsorbed. The Ga atoms which are bonded to O are shown in red. The O-bonded Ga on the surface are seen to be more elevated in the low energy region than the high.

Comparing the stable and unstable adsorption regions can reveal interesting structural patterns, which give insight into why an adsorbate would bind more strongly to one set of liquid structures than another. Side views of the most favourable snapshot in the stable region are shown in Fig. 4a and b, and the most favourable structure in the unstable region in 4c and d. Despite the ∼0.7 eV adsorption energy difference between regions, formate is adsorbed similarly through Ga–O bonds in both cases. However one can observe that the Ga atoms which formate bonds to are elevated from the rest of the surface in the stable adsorption region. This does not occur in the higher energy region which instead has these Ga recessed. While this considers only a single snapshot, the vertical (*z*) position of the formate-bonded-Ga atoms is tracked over both regions in Fig. 5. Here, one can see that the bonded Ga stay lower into the surface throughout the unstable adsorption region, whereas at least one is always elevated in the stable region. We argue that the structure with elevated Ga represents a realistic “at-temperature” snapshot, where the adsorbate has been allowed to freely interact with the dynamic metal during the AIMD run. If the structures were relaxed, as is done for static calculations of solids, this stand-out configuration of the liquid metal, which persists over an appreciable block of our sampling time, would not have been located.

The adsorption sampling for formate on Ga–In was repeated three times (See Fig. S8 to S10 and Table S1 in the ESI†), initiating the run from a different random geometry of formate each time. In all cases, we found that the average adsorption energy in the identified stable regions was very similar, with less than 0.12 eV total variation between all three runs. In each case, formate favoured a configuration bridging two Ga atoms that are slightly raised from the surface. The global average adsorption energies were also all within 0.1 eV. Slightly more variance was observed between high energy regions, with the example in Fig. 1 having an average unstable region adsorption energy of −0.63 eV, and the three replicates having averages of around −0.86 eV.

The observation of raised Ga bares some resemblance to previous reports of a single surface Ga atom being raised in a Ga–Pt alloy alloys, which has previously been implicated in activating the material to catalysis.^40^ Although the raised Ga atoms in the present case do not persist throughout the whole AIMD trajectory, our results still suggest a link between snapshots with elevated Ga and favourable adsorption of molecules.

### Multi-step surface reactions

**Fig. 6:**
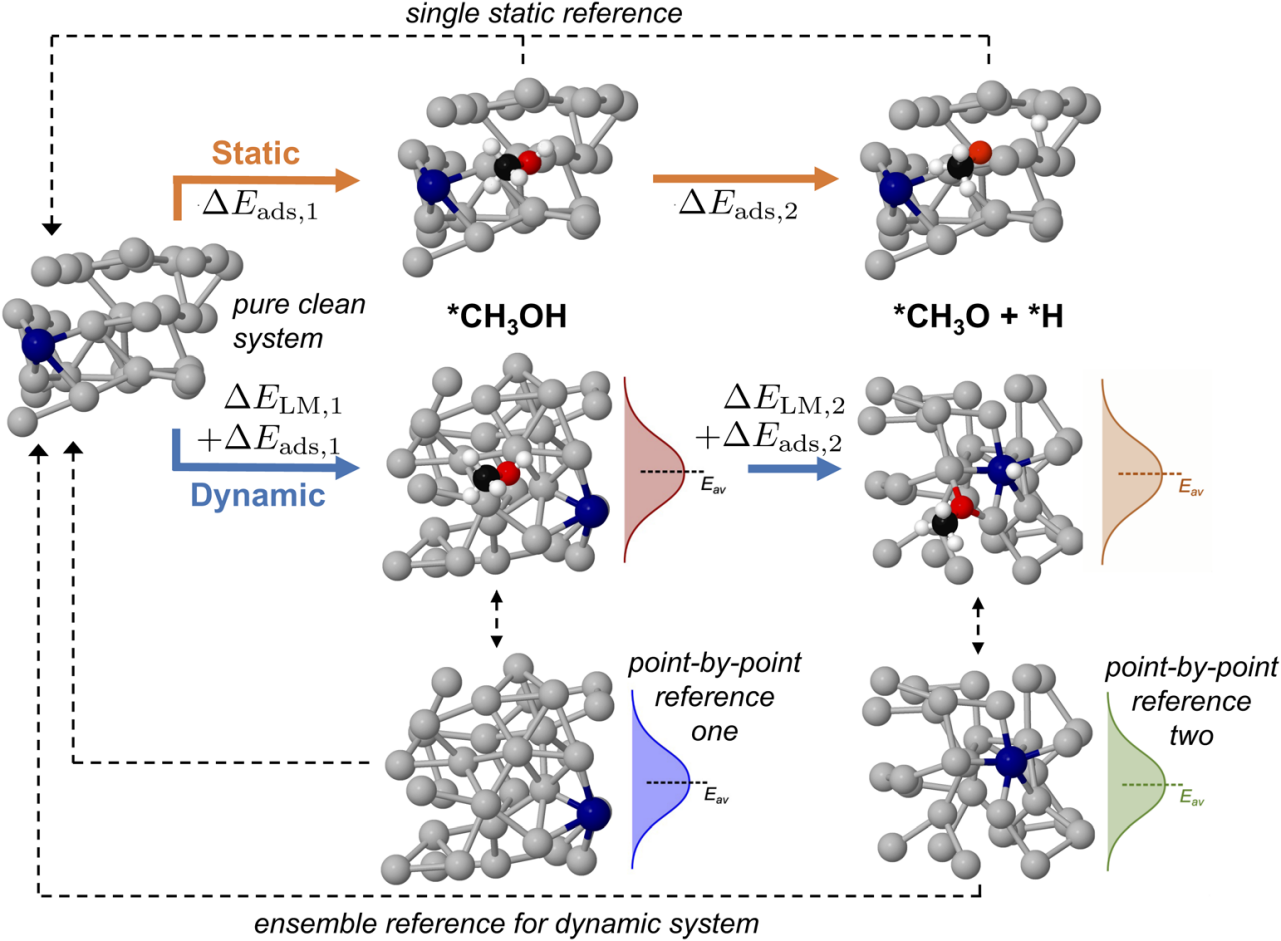
A schematic showing how an adsorption energy is traditionally calculated relative to a static reference state in a multi-step reaction pathway. For a dynamic liquid metal this is challenging, as the structure of the liquid metal changes, resulting in a different energy distribution for each intermediate. Here, we calculate a point-by-point interaction energy between liquid metal and adsorbate Δ*E*_ads_, then reference this back to the pure clean liquid metal which accounts for the energy to reorganise this liquid metal structure Δ*E*_LM_. Key: Ga-silver, Pt-blue, O-red, C-black, H-white.

Up until now we have concerned ourselves with only the information that can be gleaned from AIMD sampling of a single adsorbate on a liquid metal. However, mapping multi-step reaction processes is necessary to construct reaction energy diagrams, which are used ubiquitously in catalytic modelling.^18,20,41,42^ For pathways on a solid catalyst, this is accomplished by selecting a reference state, then calculating the energy of each subsequent reaction step relative to the energy of this reference (see static pathway in Fig. 6). However, for a liquid metal catalyst there exists an ensemble of reference structures for each adsorbed state. Each unique reference state distribution (point-by-point reference in Fig. 6) is calculated from single point energy evaluations of sampled structures with the adsorbate removed. As a result, reference states for a multi-step reaction path are more complex because the ensemble reference for the first intermediate may be different to that for the second. Fig. S11 in the ESI† shows that the average total energy of the ensemble of reference structures for successive states in the deprotonation of methanol can vary by up to 1 eV, even though the shape and form of the energy distribution stays the same. This variation can be ascribed to adsorbates influencing the structure of the liquid metal differently, thus yielding a shifted average for the total energies.

In order to ensure a consistent reference state for a multi-step reaction, we calculate the adsorbate–catalyst interaction energy using our point-by-point reference (Δ*E*_ads_ in Fig. 6). However, we also shift the energies in this reference state distribution such that the arithmetic mean is the same as that for a pure clean surface that has been allowed to propagate freely with AIMD for the same duration. This energy shift term, or "translation energy", refers to the energy cost for a pure liquid metal to reorganise around an adsorbate (Δ*E*_LM_ in Fig. 6). The translation energy shift enables comparison between states with different liquid metal geometries on a universal scale, while still allowing the point-to-point energy subtractions between adsorbate and liquid metal that are necessary to obtain adsorption energies. A detailed example of how this process is applied is given in Table S2 of the ESI.†

In the previous single-step example of formate adsorption to Ga–In, the reference state had the same average energy as the pure clean reference (differing by less than 0.05 eV), so a translation was unnecessary to model this particular process. However, in cases where an adsorbate significantly perturbs a liquid metal geometry, we argue the energy cosy to rearrangement is a realistic part of the adsorption process and should be included.

### Application to methanol oxidation on Ga–Pt

In past computational work,^10^ a 32 atom Ga–Pt model was used to study methanol oxidation and map out the reaction path. The catalyst model was an approximately 9 × 9 Å surface slab, the structure of which was generated by an AIMD run, then relaxed to perform adsorption studies. Following past literature,^10,43^ the methanol oxidation reaction is treated as a set of successive deprotonation steps after methanol has adsorbed to the surface:

3 CH_3_OH → *CH_3_OH

4 *CH_3_OH → *CH_3_O + H*

5 *CH_3_O + H* → *CH_2_O + 2H*

6 *CH_2_O + 2H* → *CHO + 3H*

7 *CHO + 3H* → *CO + 4H*

Alternative pathways have also been proposed depending on the electrochemical conditions,^44,45^ but these are not considered here.

**Fig. 7:**
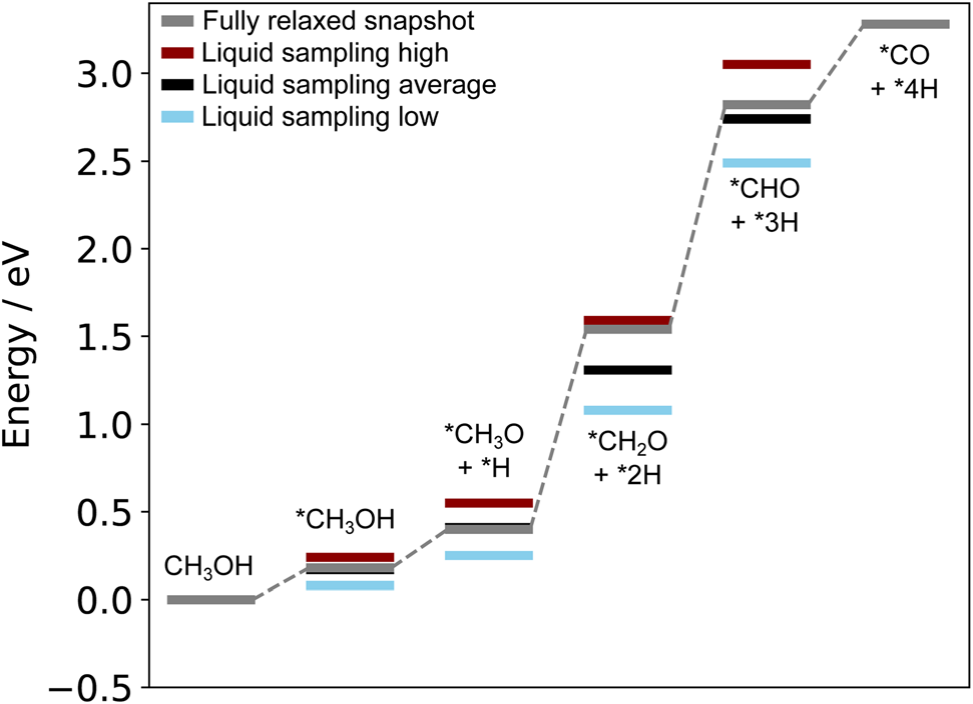
Reaction energy diagram for the oxidation of methanol on liquid Ga–Pt at 318 K. The grey line denotes adsorption energies on a fully relaxed structure of the liquid metal. The other lines indicate the average (black), unstable (red), and stable (blue) adsorption energy regions when AIMD sampling is performed.

The structures reported by Rahim *et al.*^10^ on Ga–Pt served as a starting point for us to run the liquid metal sampling technique on each of these intermediates. We also performed fully relaxed snapshot calculations to compare with those from our liquid metal sampling. The calculated reaction energy diagram for each treatment is shown in Fig. 7. For liquid metal sampling, the stable (blue) and unstable (red) adsorption region averages are shown in addition to the global average (black). Plots tracking the adsorption energy over time during liquid metal sampling are available in Fig. S12 to S18 in the ESI.†

From Fig. 7, it is seen that the adsorption energy for the fully relaxed structures tends to sit somewhere within the distribution described by liquid metal sampling. This value is generally close to the average of the liquid metal sampling, but deviates in some cases (*e.g.* the *CH_2_O + *2H state, where it is close to the high energy average). It should be noted that the absolute energies of the intermediates and reference state are both raised by about the same amount for the at-temperate sampled structures compared to the relaxed. This suggests a systematic shift to the sampled energies, thus producing an adsorption energy that is consistent with full relaxation. However, the liquid metal sampling gives a distribution of energies with gaps between the high and low energy regions ranging from 0.16 to 0.56 eV. We suggest that the fully relaxed adsorption energies are effectively “drawn” from the at-temperature range, depending on what specific snapshot is chosen for relaxation. This is statistically consistent with most (but not all) fully relaxed adsorption energies sitting close to the global average for sampling. We also note that the relaxed structures pathway reported here differs from that originally calculated by Rahim *et al.*,^10^ with the adsorption energies here being shifted between 0.4 to 0.6 eV higher in energy.

**Fig. 8:**
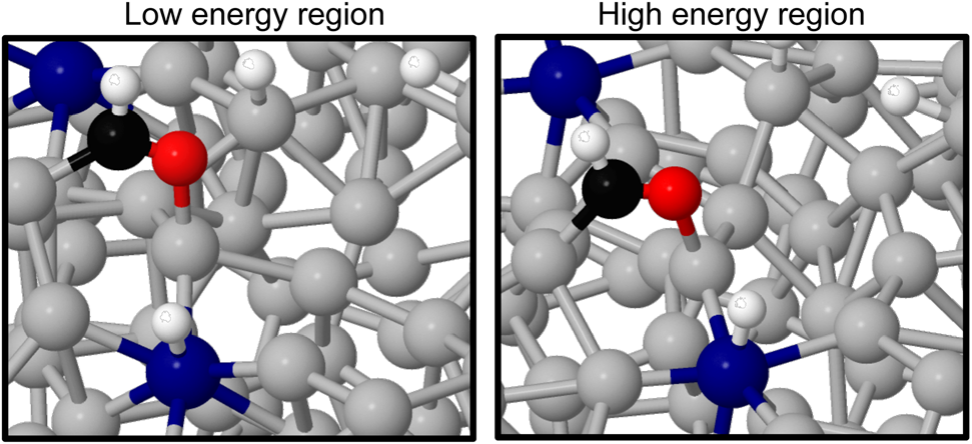
A comparison of structures from the stable (left) and unstable (right) adsorption regions for liquid metal sampling of the *CHO + *3H state. The most pronounced difference is the elevation of Ga atoms underneath the adsorbates. Key: Ga-silver, Pt-blue, O-red, C-black, H-white.

As the methanol oxidation pathway progresses, we observe an increased gap between the sampled stable and unstable adsorption regions. This can be attributed to the fact that more species exist on the Ga–Pt surface in the later reaction steps, giving rise to more variance in the adsorption energy as the liquid metal naturally rearranges. Closer inspection of the structures of the *CHO + *3H state (Fig. 8), with the largest energy gap between the high and low energy regions, shows that the Ga atoms bonding to *CHO are elevated in the low energy region but more recessed in the high. Indeed, the Ga bonded directly to C is 0.75 Å further above the surface in the stable adsorption region than the unstable. As was the case for the Ga–In example, this once again suggests that liquid metals have the ability to more readily adsorb species by spontaneously extending under-coordinated atoms from the surface as a part of their dynamic motion at temperature.

The energy cost associated with liquid metal rearrangement in the presence of adsorbates, given by the translation energy described in the Mutli-step surface reactions section, also increases along the pathway. The translation energies are given in Table S2 in the ESI,† and rise from 0.14 eV for the *CH_3_OH state, up to 1.15 eV for the *CHO + *3H state. This suggests that it becomes increasingly unfavourable for the liquid metal surface to accommodate surface-adsorbed species as they dissociate and take up more sites. Therefore, large parts of the energetic cost to adsorption can be attributed to the structural change in the liquid metal, rather than the new bonds formed to the adsorbate itself. Perhaps most notably, we were unable to sample stable geometries for the *CO + 4H* state because H_2_ spontaneously formed and desorbed from the surface during the AIMD simulation. We suggest this state would not be stable at-temperature, and H desorption would occur before it could be reached. Overall, this indicates that the pathway tested in Fig. 7 would be unlikely to occur with a realistic dynamic liquid metal.

**Fig. 9:**
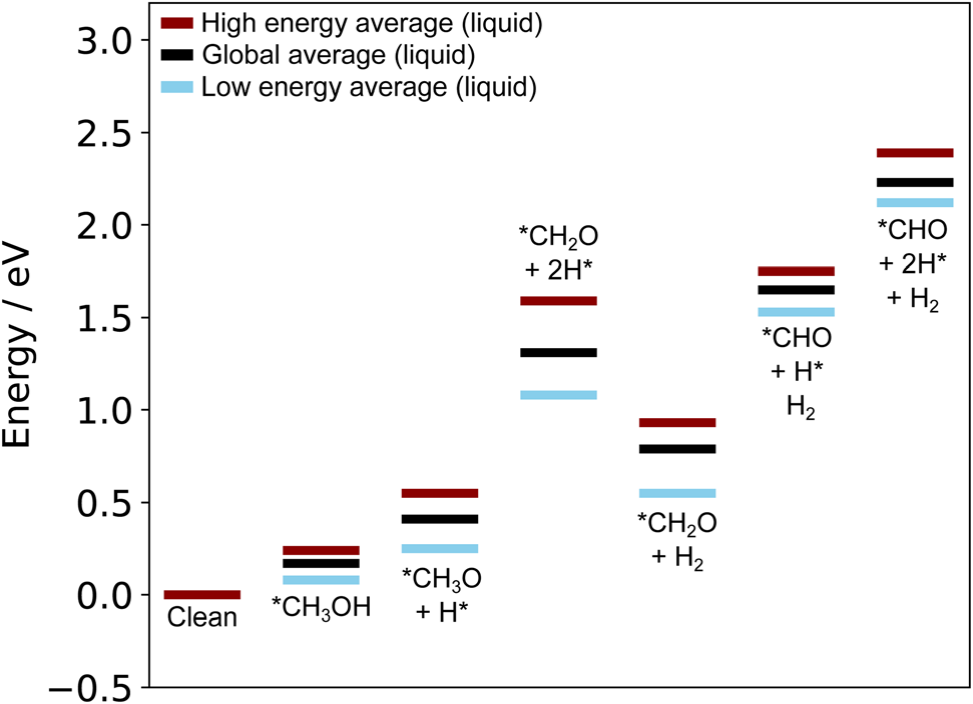
A reaction energy diagram generated using the liquid metal sampling technique which shows the methanol dehydrogenation pathway on Ga–Pt when surface adsorbed H are allowed to desorb part-way through the reaction. The liquid metal sampling technique indicates early desorption of H is a favourable processes to reduce the energy cost to reordering the liquid metal around adsorbates. This effect is not captured by pure static calculations.

Due to the instability of states with many H on the surface, we concluded that the adsorbed H produced by methanol dehydrogenation are likely to leave the surface part-way through the original pathway proposed above (either as H_2_ or to solution, depending on the electrochemical conditions).^46^ We examined the thermodynamics of this proposed process by considering moving from the *CH_2_O + 2H* state to a *CH_2_O + H_2_ state, where the H atoms have combined and desorbed from the surface. A reaction energy diagram calculated with liquid metal sampling for this proposed alternative pathway is shown in Fig. 9. Comparing this to the original pathway in Fig. 7, we see that the large uphill steps to remove the final two H from methanol are much lower in energy. Interestingly, the relative energy difference between the final three states in the pathway remains similar to the original. However, all of these states are shifted lower in energy due to the more stable *CH_2_O + H_2_ state, which lowers the overall energy cost to the pathway. This pathway minimises the adsorbate disruption to the dynamic liquid metal surface, and allows it to adopt more favourable configurations (see the translation energies in Table S2 in the ESI†). For instance, a large drop in the translation energy from 1.03 eV in the *CH_2_O + 2H* state to 0.35 eV in the *CH_2_O + H_2_ state is observed, and this can directly account for the downhill step on the energy diagram.

Therefore, liquid metal sampling suggests that the removal of H from the surface somewhere along the methanol oxidation path would be favourable, and is likely a more realistic scenario than what could be observed with only static adsorption energy calculations. Future work could also consider alternative electrochemical pathways for methanol oxidation on this interesting catalyst, including understanding the role of hydroxide and solvent.^44,45^

## Conclusion

Herein we have proposed a new approach for more accurately accounting for adsorption of species to liquid metal catalysts. Our technique allows for dynamic sampling of the adsorbate and liquid metal, giving a range of possible adsorption energies that represent an at-temperature dynamic catalyst. We find that our AIMD sampling can locate different binding configurations for adsorbates that are persistently higher or lower in energy than those that are found from traditional static relaxation calculations. This indicates static calculations are not able to find the most stable nor the most catalytically relevant structures, and implicates the immediate applicability of this methodology for studying these complex systems more rigorously.

All the calculations reported here represent a liquid metal interface with the gas phase. Yet, should computational resources allow, the sampling technique could also be applied with the inclusion of a solvent. This could prove particularly useful for studying reactions where the solvent either stabilises intermediates or is directly involved in the reaction.^47^

The liquid metal sampling technique eliminates the problem of choosing single representative surface snapshots to study catalytic processes, and instead gives insight into which surface features stabilise and destabilise an adsorbate (*e.g.* raised Ga atoms in the case of formate and methanol derivatives). This may also be used to infer the most likely geometries for reaction in a mechanistic pathway. For instance, adsorption processes are more likely to occur to reach structures in low energy regions of sampling, whereas desorption or conversion processes may be more likely from high energy structures.

Ultimately, the calculation of reaction barriers linking thermodynamic states in a mechanistic profile would be desirable. However, this is challenging at present as a reaction event could in principle occur from any sampled structure in one state to any sampled structure in another. Without microkinetic modelling it would be difficult to ascertain to what degree different possible conversion processes contributed to the overall rate of a reaction step. Going forwards, we aim to investigate this problem using metadynamic sampling^32,48^ to connect different thermodynamic states on liquid metal catalysts.

## Data availability

We include a large body of our computational data in the ESI† of this manuscript. The scripts used to perform our analyses can be found at: www.github.com/CharlieRuffman/AdsorptionSampling.

## Author contributions

C. Ruffman and N. Gaston conceived the idea for this study, and designed the experiments. C. Ruffman performed the calculations. K. G. Steenbergen and A. L. Garden helped with analysis and interpretation of the data. C. Ruffman wrote the manuscript and all authors assisted with editing, analysis, and interpretation.

## Conflicts of interest

There are no conflicts to declare.

## Supplementary Material

SC-015-D3SC04416E-s001
