# Supplementary material for: Dynamic sampling of liquid metal structures for theoretical studies on catalysis
Source: Chem Sci. 2023 Nov 29;15(1):185–94. doi: 10.1039/d3sc04416e (PMC10732005; doi:10.1039/d3sc04416e)
Supplement: SC-015-D3SC04416E-s001 [file SC-015-D3SC04416E-s001.pdf]

Electronic Supplementary Material (ESI) for Chemical Science.
This journal is © The Royal Society of Chemistry 2023

Supporting information for: Sampling liquid metal structures for theoretical studies on catalysis

Charlie Ruffman,[†] Anna Garden,[‡] Krista G. Steenbergen,[¶] and Nicola Gaston[*,†]

†*MacDiarmid Institute for Advanced Materials and Nanotechnology and and Department of Physics, University of Auckland, Private Bag 92019, Auckland, New Zealand*

‡*MacDiarmid Institute for Advanced Materials and Nanotechnology and Department of Chemistry, University of Otago, P.O. Box 56, Dunedin 9054, New Zealand*

¶*MacDiarmid Institute for Advanced Materials and Nanotechnology and School of Chemical and Physical Sciences, Victoria University of Wellington, PO Box 600, Wellington 6140, New Zealand*

E-mail: n.gaston@auckland.ac.nz

Computational details

All electronic structure calculations reported in the main text were performed using the Vienna *Ab initio* Simulation Package (VASP).[S1] Electronic energies were obtained by iterative diagonalisation of the Kohn-Sham Hamiltonian. Electrons were described using a plane-wave basis set with a kinetic energy cut-off of 450 eV for Ga-In liquid metal systems, and 400 eV for Ga-Pt systems. These values were chosen in accordance with past work on each of the different systems.[S2,S3] For Ga-In systems, Perdew–Burke–Ernzerhof for solids (PBEsol)[S4] was used as the exchange correlation functional, which has previously been demonstrated to capture Ga systems well.[S2,S5,S6] Ga-Pt was calculated using the original Perdew–Burke–Ernzerhof functional,[S7] in order to keep consistency with past work.[S3] Methfessel–Paxton (order one, 0.05 eV width) smearing was applied to the Kohn-Sham states in order to aid convergence. The energy convergence threshold for electronic optimisation was set to 10^{-5} eV. Reciprocal space was sampled using 3×2×1 Monkhorst-pack k-points for Ga-In, and 4×4×1 for Ga-Pt. The projector augmented wave method was used to account for core electrons.[S8,S9]

Ab initio molecular dynamics (AIMD) calculations were performed within the NVT ensemble, using a 1 fs time step. The short time step was required due to the inclusion of light elements (e.g. H) in adsorbates on the liquid metal surfaces. A Verlet algorithm was used to solve the equations of motion, with the temperature controlled by coupling to a Nosé–Hover thermostat.[S10]

Scripts

The codes used for setting up and processing bulk VASP calculations are available on GitHub at: `https://github.com/CharlieRuffman/AdsorptionSampling.git`. These codes are written to work with the atomic simulation environment. See the README file for more information on how to run these scripts.

Extended sampling for the $^*CH_2O + 2\,H^*$ state

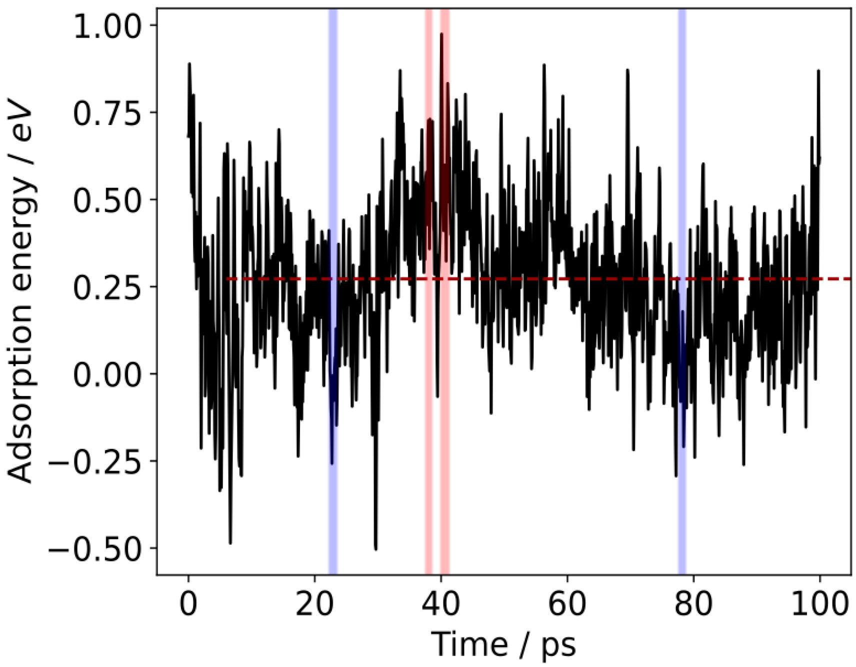

Figure S1: Adsorption energy sampling plot for the $^*CH_2O + 2\,H^*$ state on Ga-Pt extended from 40 ps to 100 ps. The high and low energy regions identified in the first 40 ps of sampling time are not exceeded (though are relocated) at the longer 100 ps time window.

Resolutions for mirror MD

To explore the effect of different resolutions for the mirror MD calculations required for the reference state, the following tests were performed on a 10 ps window within the sampling for formate adsorbing to Ga-In. The resolutions sampled here span from taking a reference state every 10 time-steps (i.e. 10 fs) to 100 time-steps.

The maximum and minimum adsorption energy peaks, as well as the average adsorption energy, all stay highly consistent at resolutions from 10 to 80 fs. It is only at a resolution of 100 fs where a narrow maximum energy peak is seen to decrease by about 0.1 eV relative to more precise resolutions. Although the resolution can be adjusted to fit the user's needs, these data suggest that a resolution of 80 fs is completely sufficient if one wishes to consider

regions of high or low adsorption energy (e.g. 0.5 ps wide). One may also only expect relatively small errors in the maximum/minimum energy (e.g. around 0.1 eV) going up to resolutions of 100 fs.

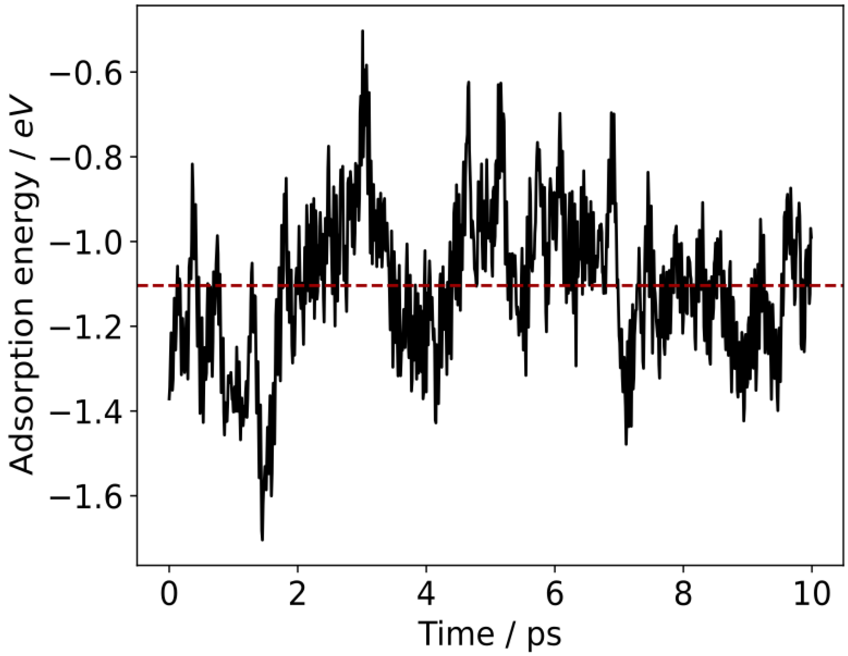

Figure S2: 10 fs resolution. The average energy is

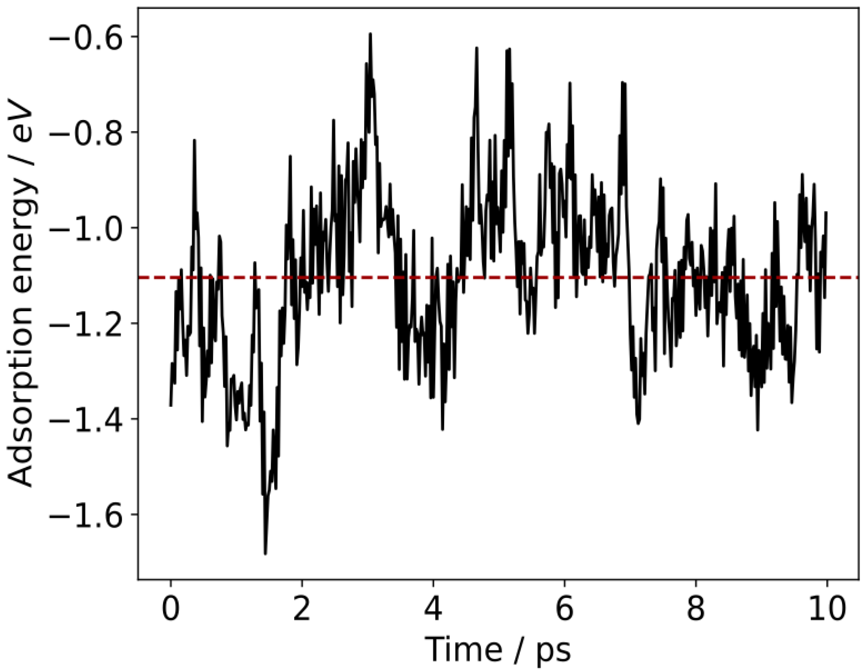

Figure S3: 20 fs resolution. The average adsorption energy is -1.10 eV.

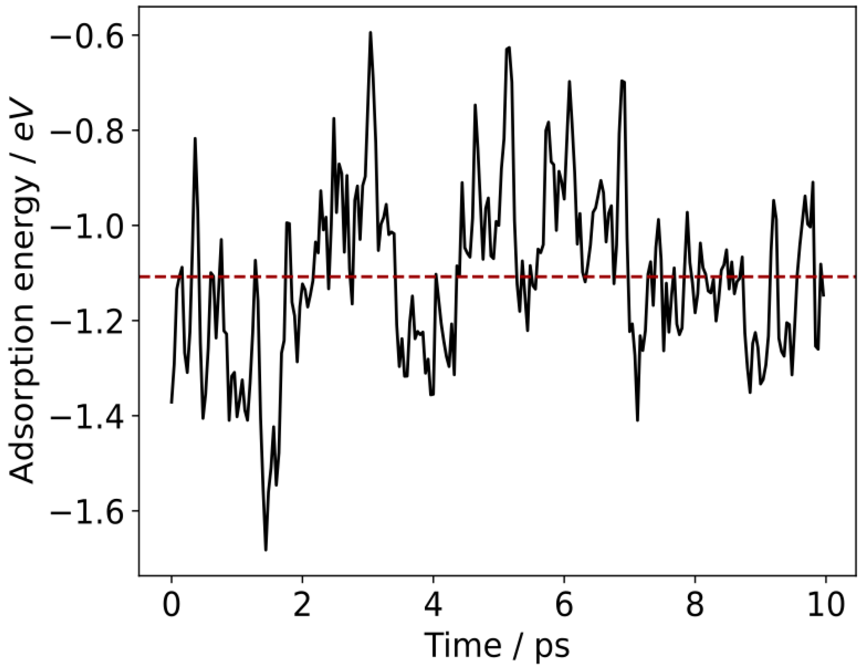

Figure S4: 40 fs resolution. The average adsorption energy is -1.11 eV.

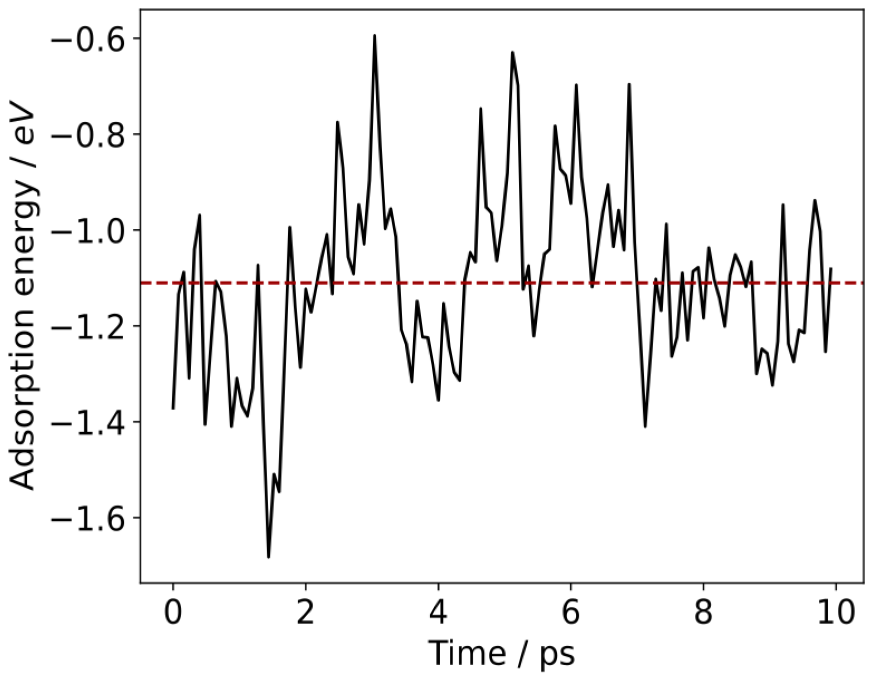

Figure S5: 80 fs resolution. The average adsorption energy is -1.11 eV.

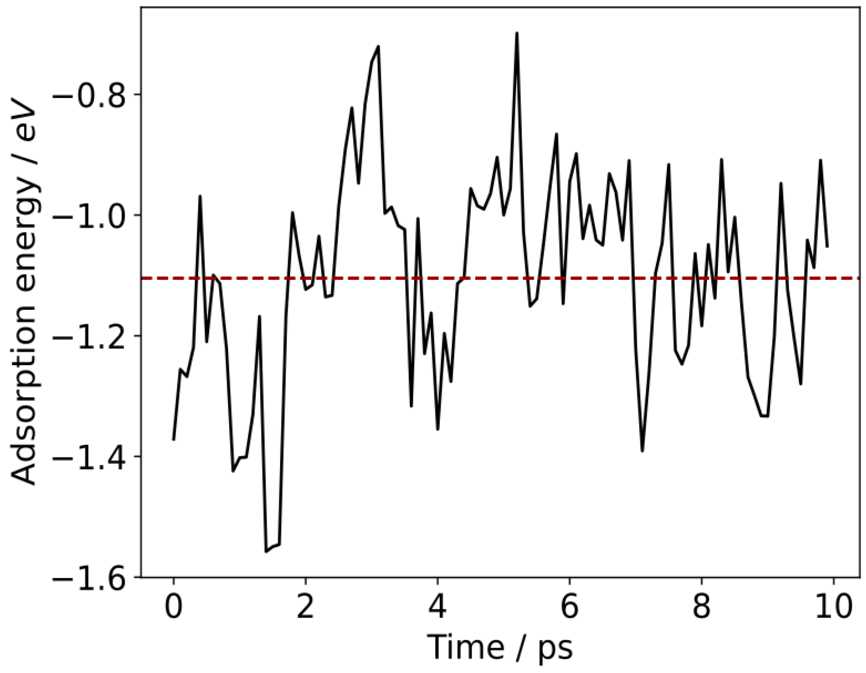

Figure S6: 100 fs resolution. The average adsorption energy is -1.10 eV.

Mean squared displacement of metal atoms and adsorbate

From Figure S7, it can be seen that the mean square displacement of formate adsorbed on Ga-In is much larger for the adsorbate atoms themselves than the metal atoms below. This suggests that adsorbate atoms move more, and more quickly, than the structure of the metal below.

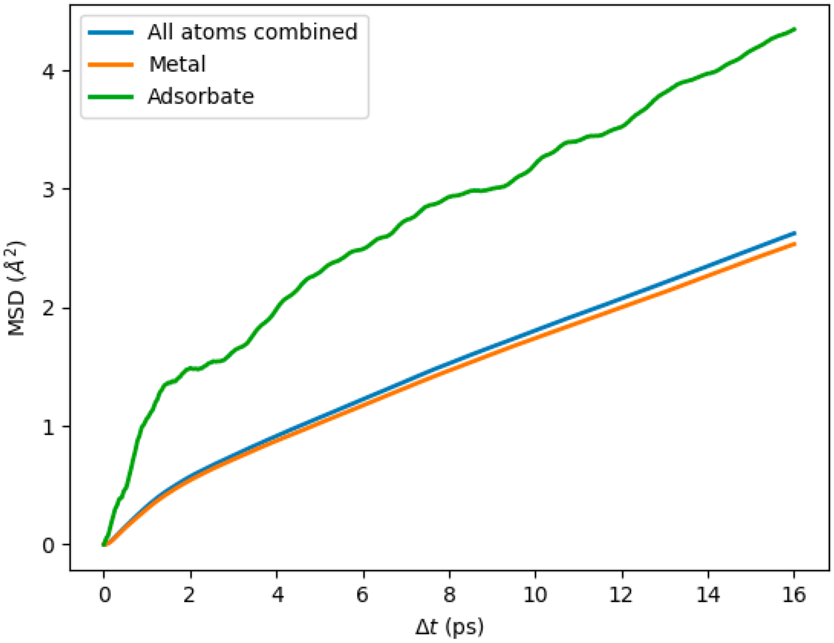

Figure S7: Mean squared displacement (MSD) of metal atoms in Ga-In compared to that for a formate adsorbate sampled on the surface over 40 ps. The MSD is shown calculated over different time intervals (from 0 to 16 ps) across the sampling duration in order to remove any effect of bias from the starting configuration.

Repeat trials for formate adsorption to Ga-In

Three repeat samples for formate adsorption on Ga-In were conducted, each time initiating the run by placing formate in a different location from the example in the main text. A half-size surface model of 66 Ga and 6 In was used in order to conserve computational cost. The model was still six-layers in the z-direction and maintained the same proportion of In at the surface. The energy sampling plots are shown in Figures S8 to S10, and the energies of each different system are compared in Table S1. It is observed that, despite very different starting configurations that yield disparate adsorption energies, there is general agreement between the sampled low energy regions and the global average adsorption energies. This suggests a degree of consistency in which structures are located, regardless of the chosen starting configuration.

Table S1: Energies for repeat trials of liquid metal sampling of formate on Ga-In at 450 K. All energies are given in eV.

System	Average of low energy window / eV	Global average / eV	Average of high energy window / eV
Main paper Ga-In	-1.28	-0.99	-0.63
Replicate 1	-1.16	-0.99	-0.81
Replicate 2	-1.30	-1.07	-0.85
Replicate 3	-1.23	-1.06	-0.94

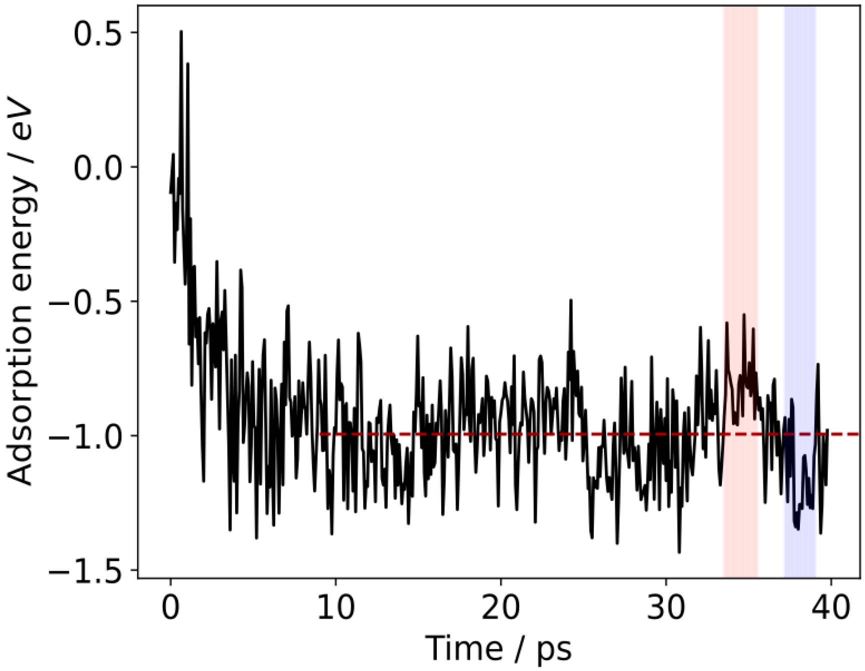

Figure S8: Adsorption energy sampling plot for replicate 1 of formate adsorption on Ga-In at 450 K.

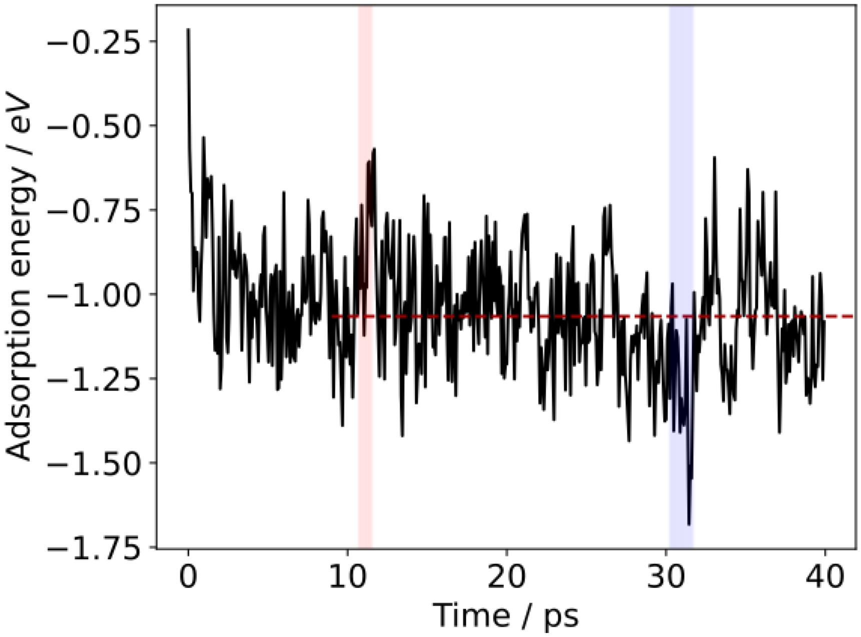

Figure S9: Adsorption energy sampling plot for replicate 2 of formate adsorption on Ga-In at 450 K.

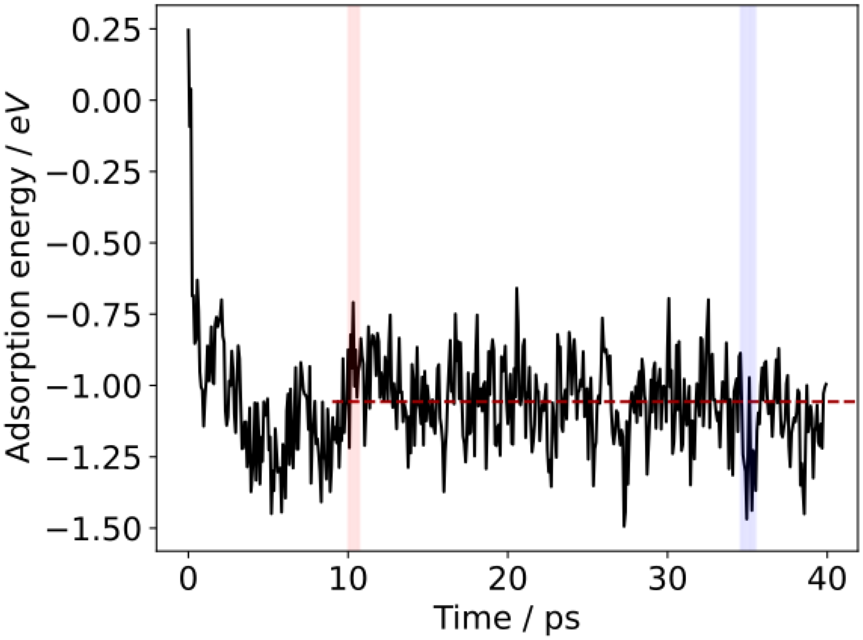

Figure S10: Adsorption energy sampling plot for replicate 3 of formate adsorption on Ga-In at 450 K.

Reference states in a multi-step pathway

Figure S11 shows the energy distributions of several reference states taken from the multi-step pathway for the oxidation of methanol. All of these surfaces are "clean," in that the adsorbate is not present, but the mean energies of these systems is still shown to differ. This is likely a result of the adsorbate influencing the surface structure of the liquid metal. Therefore, to ensure a consistent reference for all reaction steps and capture possible energy differences from surface rearrangement, the energies of all reference states was translated such that the mean matches that of the "pure" clean system with no adsorbate present (Figure S11a). Note that this does not change the shape of the distribution or the sampled structures, it merely removes any systematic shift in the energy of the Ga-Pt surface due to adsorbates being present. The data involved in these translations is shown in Table S2.

Table S2: Average total energies for the adsorbed and reference states involved in the multi-step path for methanol oxidation, alongside the amount by which the reference state energies need to be translated by to have the same average energy as the pure clean system. Note that the energy of the atoms in methanol (i.e. methanol in a box) is -30.21 eV. All energies shown are in eV.

System	Average E(reference)	Translation amount	Average E(adsorbed)	Average ΔE_{ads}	Average ΔE_{ads} (translated)
Pure clean GaPt	-92.14	–	–	–	–
$^*CH_3OH$	-92.00	-0.14	-122.17	0.03	0.17
$^*CH_3O + H^*$	-91.45	-0.68	-121.86	-0.27	0.41
$^*CH_2O + 2\,H^*$	-91.11	-1.03	-121.04	0.28	1.31
$^*CHO + 3\,H^*$	-90.99	-1.15	-119.61	1.59	2.74
$^*CH_2O + H_2$	-91.79	-0.35	-114.79	0.44	0.79
$^*CHO + H^* + H_2$	-92.11	-0.02	-113.93	1.62	1.65
$^*CO + 2\,H^* + H_2$	-91.92	-0.21	-113.34	2.02	2.23

It is observed that the average total energy of the reference states grows increasingly

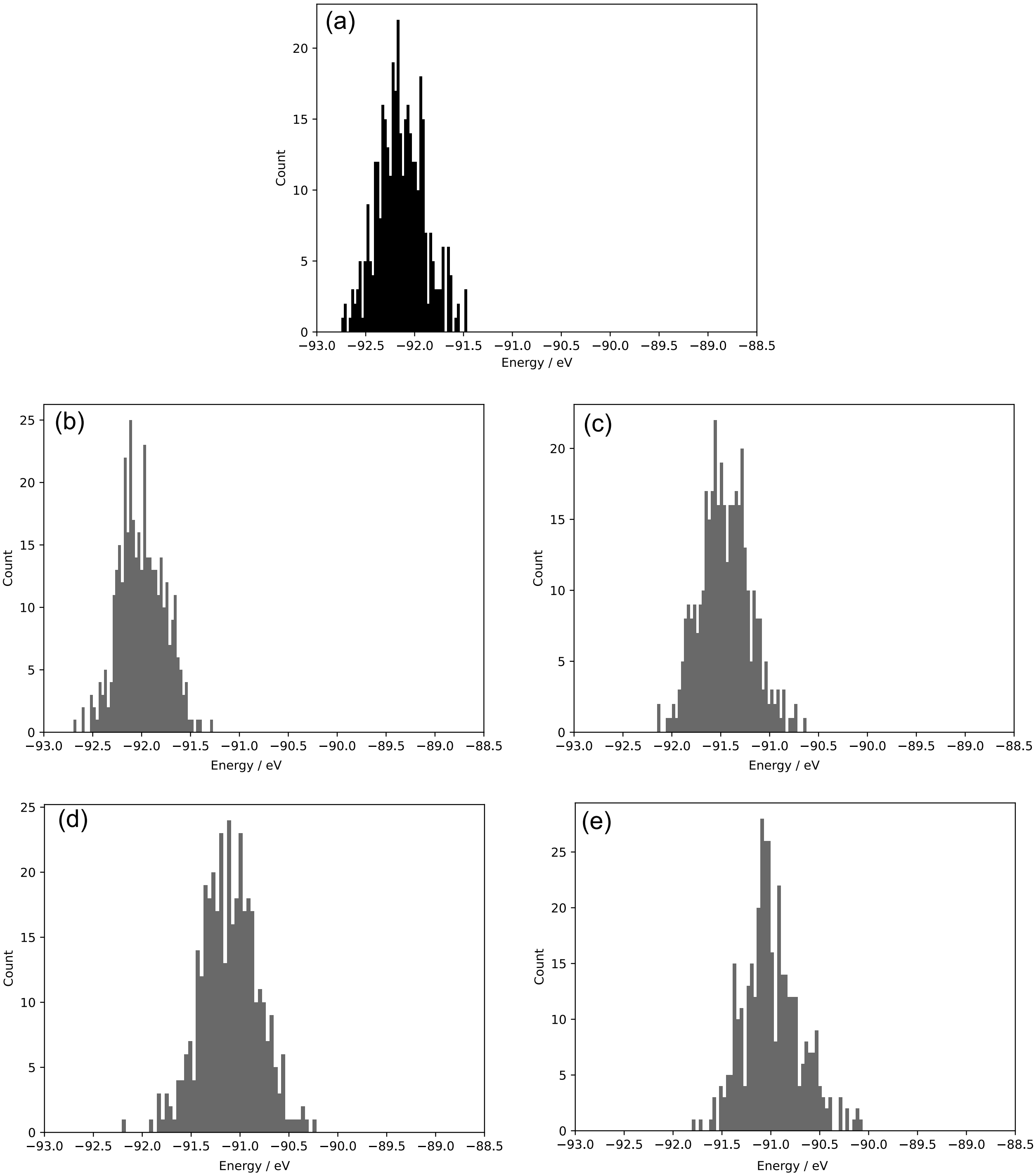

Figure S11: Energy distributions of (a) the "pure" clean Ga-Pt structure ($E_{\mathrm{mean}} = -92.17$), and (b-f) the adsorbate-removed reference states for the multi-step methanol oxidation pathway. The states are as follows: (b) $^{*}CH_3OH$ ($E_{\mathrm{mean}} = -91.97$), (c) $^{*}CH_3O + H^{*}$ ($E_{\mathrm{mean}} = -91.37$), (d) $^{*}CH_2O + 2\,H^{*}$ ($E_{\mathrm{mean}} = -90.93$), (e) $^{*}CHO + 3\,H^{*}$ ($E_{\mathrm{mean}} = -90.88$),

higher (less stable) compared to the pure clean Ga reference as the methanol oxidation pathway advances. This is likely a result of a greater number of separate surfaces species being present as the reaction progresses, therefore disrupting the structure of the GaPt. We believe these increases in energy represent a real energetic cost to the reaction occurring on the surface, which would not be captured by traditional modelling of snapshots at energy minima.

Adsorption energy sampling plots for states in free energy diagrams

The following figures show the adsorption energy, as sampled over time, for all the different states that are presented in free energy diagrams for the methanol oxidation reaction. Pathway 1 includes the states shown in Figure 6 in the main text, and Pathway 2 the new states covered in Figure 8.

It can be noted that in some cases (e.g. Figure S12 and S13), the adsorption energy sampling starts from a geometry with a favourable adsorption energy, but then rises in energy. This suggests the optimised structure that these runs were initiated from would not actually stably exist at-temperature. In other cases (e.g. Figure S14), the starting geometry appears to be far from the most favourable liquid metal arrangement, and the adsorption energy sampling is able to locate a more stable configuration.

Pathway 1

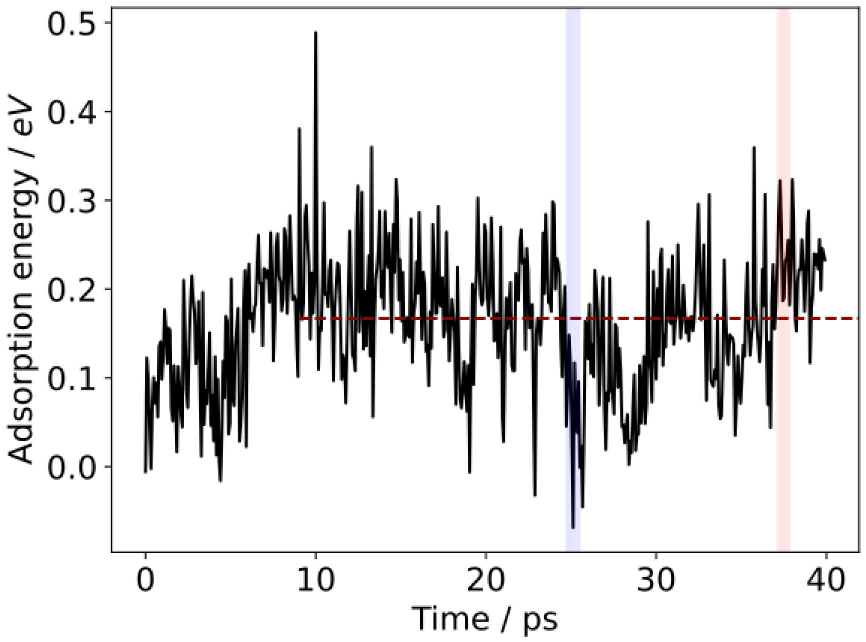

Figure S12: Adsorption energy sampling plot for the $^{*}CH_3OH$ state on Ga-Pt.

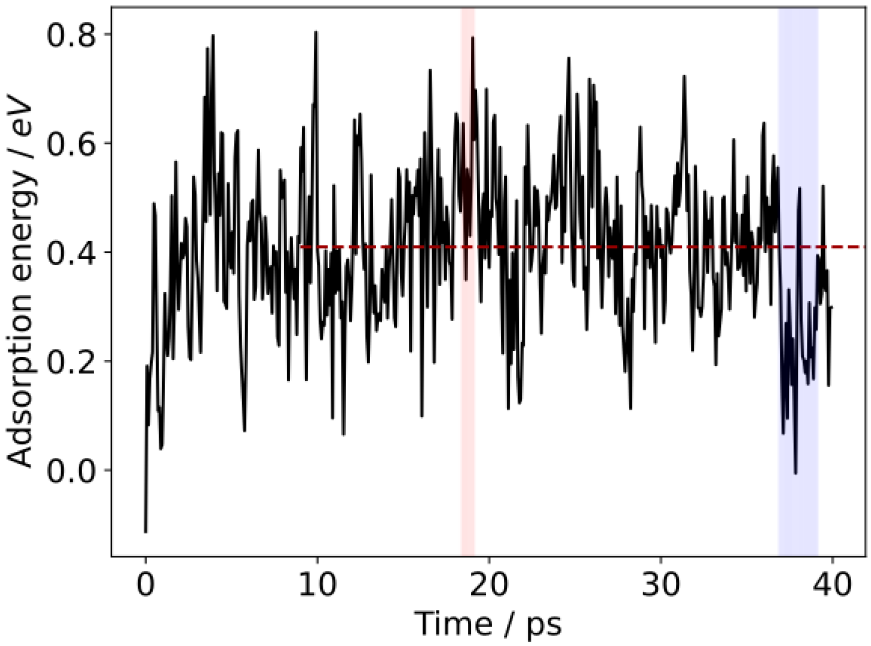

Figure S13: Adsorption energy sampling plot for the $^*CH_3O + {}^*H$ state on Ga-Pt.

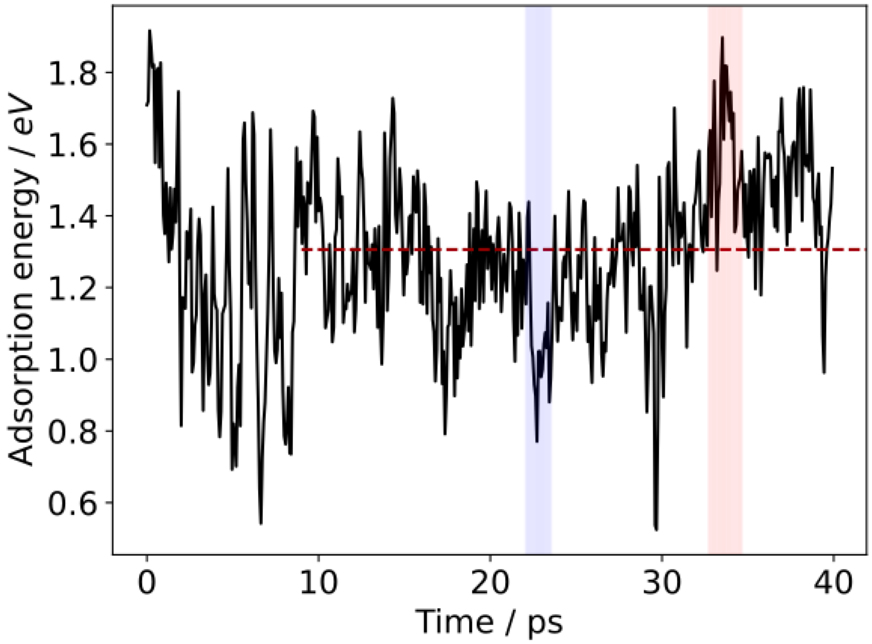

Figure S14: Adsorption energy sampling plot for the $^*CH_2O + 2\,H^*$ state on Ga-Pt.

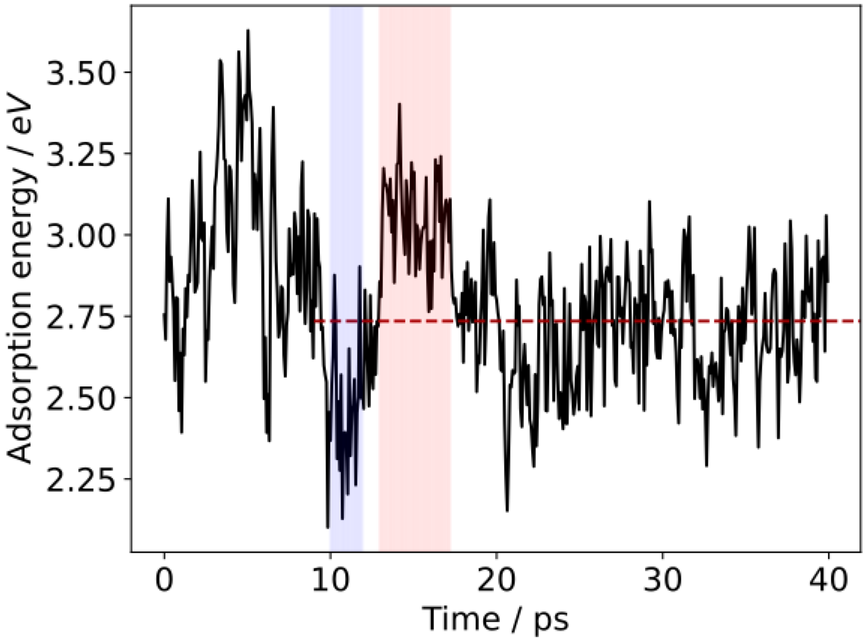

Figure S15: Adsorption energy sampling plot for the $^{*}CHO + 3\,H^{*}$ state on Ga-Pt.

Pathway 2

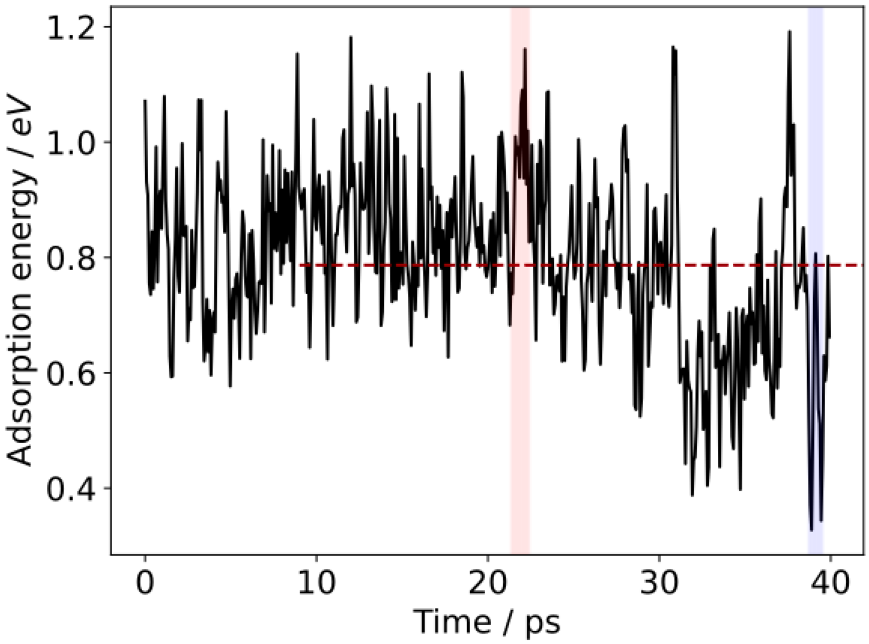

Figure S16: Adsorption energy sampling plot for the $^*CH_2O + H_2$ state on Ga-Pt.

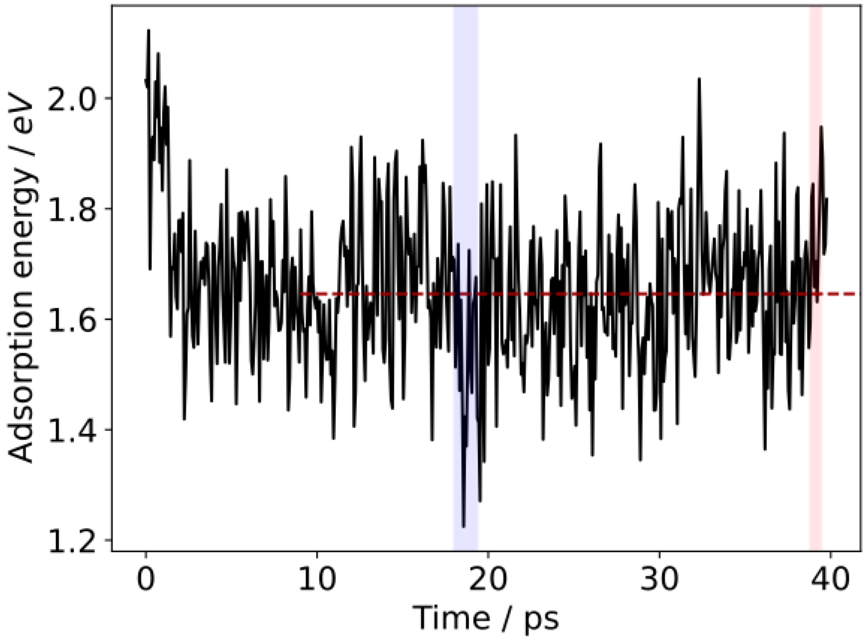

Figure S17: Adsorption energy sampling plot for the $^*CHO + {}^*H + H_2$ state on Ga-Pt.

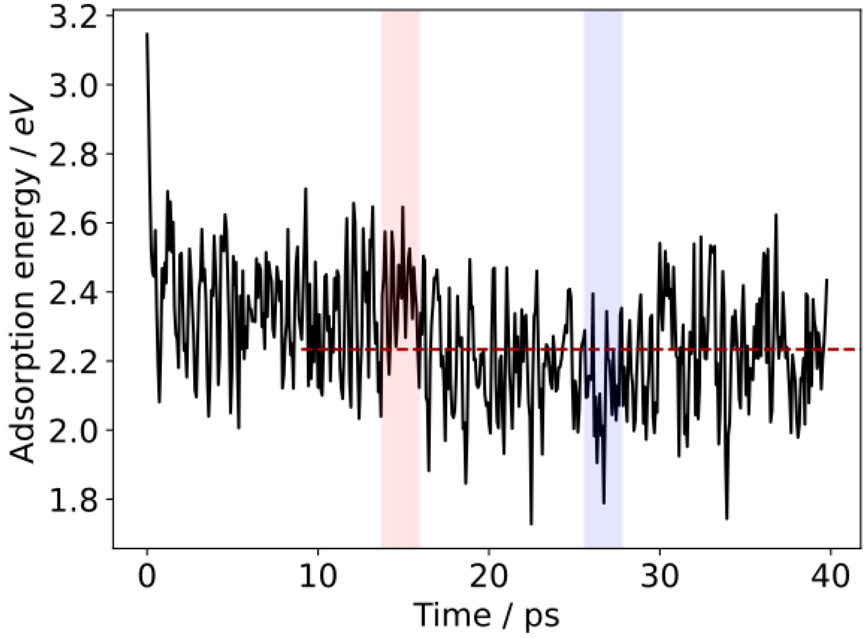

Figure S18: Adsorption energy sampling plot for the $^*CO + 2\,H^* + H_2$ state on Ga-Pt.

References

(S1) Kresse, G.; Furthmüller, J. *Comput. Mater. Sci.* **1996**, *6*, 15–50.

(S2) Ruffman, C.; Lambie, S.; G. Steenbergen, K.; Gaston, N. *Physical Chemistry Chemical Physics* **2023**, *25*, 1236–1247.

(S3) Rahim, M. A.; Tang, J.; Christofferson, A. J.; Kumar, P. V.; Meftahi, N.; Centurion, F.; Cao, Z.; Tang, J.; Baharfar, M.; Mayyas, M.; Allioux, F.-M.; Koshy, P.; Daeneke, T.; McConville, C. F.; Kaner, R. B.; Russo, S. P.; Kalantar-Zadeh, K. *Nature Chemistry* **2022**, *14*, 935–941.

(S4) Perdew, J. P.; Ruzsinszky, A.; Csonka, G. I.; Vydrov, O. A.; Scuseria, G. E.; Constantin, L. A.; Zhou, X.; Burke, K. *Physical Review Letters* **2008**, *100*, 136406.

(S5) Lambie, S.; Steenbergen, K. G.; Gaston, N. *Nanoscale Advances* **2021**, *3*, 499–507.

(S6) Steenbergen, K. G.; Gaston, N. *Chemical Communications* **2019**, *55*, 8872–8875.

(S7) Perdew, J. P.; Burke, K.; Ernzerhof, M. *Physical Review Letters* **1996**, *77*, 3865–3868.

(S8) Kresse, G.; Joubert, D. *Physical Review B* **1999**, *59*, 1758.

(S9) Blöchl, P. E. *Physical Review B* **1994**, *50*, 17953–17979.

(S10) Nosé, S. *The Journal of Chemical Physics* **1984**, *81*, 511–519.
